# Appropriate Handling, Processing and Analysis of Blood Samples Is Essential to Avoid Oxidation of Vitamin C to Dehydroascorbic Acid

**DOI:** 10.3390/antiox7020029

**Published:** 2018-02-11

**Authors:** Juliet M. Pullar, Simone Bayer, Anitra C. Carr

**Affiliations:** Department of Pathology and Biomedical Science, University of Otago, Christchurch, P.O. Box 4345, Christchurch 8140, New Zealand; juliet.pullar@otago.ac.nz (J.M.P.); simone.bayer@otago.ac.nz (S.B.)

**Keywords:** vitamin C, ascorbate, dehydroascorbic acid, whole blood, plasma, EDTA, heparin, HPLC with electrochemical detection, pneumonia, cancer, diabetes, critically ill

## Abstract

Vitamin C (ascorbate) is the major water-soluble antioxidant in plasma and its oxidation to dehydroascorbic acid (DHA) has been proposed as a marker of oxidative stress in vivo. However, controversy exists in the literature around the amount of DHA detected in blood samples collected from various patient cohorts. In this study, we report on DHA concentrations in a selection of different clinical cohorts (diabetes, pneumonia, cancer, and critically ill). All clinical samples were collected into EDTA anticoagulant tubes and processed at 4 °C prior to storage at −80 °C for subsequent analysis by HPLC with electrochemical detection. We also investigated the effects of different handling and processing conditions on short-term and long-term ascorbate and DHA stability in vitro and in whole blood and plasma samples. These conditions included metal chelation, anticoagulants (EDTA and heparin), and processing temperatures (ice, 4 °C and room temperature). Analysis of our clinical cohorts indicated very low to negligible DHA concentrations. Samples exhibiting haemolysis contained significantly higher concentrations of DHA. Metal chelation inhibited oxidation of vitamin C in vitro, confirming the involvement of contaminating metal ions. Although EDTA is an effective metal chelator, complexes with transition metal ions are still redox active, thus its use as an anticoagulant can facilitate metal ion-dependent oxidation of vitamin C in whole blood and plasma. Handling and processing blood samples on ice (or at 4 °C) delayed oxidation of vitamin C by a number of hours. A review of the literature regarding DHA concentrations in clinical cohorts highlighted the fact that studies using colourimetric or fluorometric assays reported significantly higher concentrations of DHA compared to those using HPLC with electrochemical detection. In conclusion, careful handling and processing of samples, combined with appropriate analysis, is crucial for accurate determination of ascorbate and DHA in clinical samples.

## 1. Introduction

The role of vitamin C (ascorbate) in health and disease has been widely studied since its discovery in the 1930s [1]. Ascorbate is the primary water-soluble antioxidant in plasma and a major non-enzymatic antioxidant in tissues; it can protect many important biomolecules from oxidation as well as regenerate specific molecules, such as vitamin E and tetrahydrobiopterin [2,3]. It is ascorbate’s enzyme cofactor activity, however, that is likely to be its most significant function in vivo, with the list of biosynthetic and regulatory enzymes for which ascorbate is an essential cofactor growing [4,5]. Ascorbate is a cofactor for the metalloenzymes required for collagen tertiary structure stabilisation, transport of fatty acids into mitochondria for the generation of metabolic energy, and synthesis of catecholamine neurotransmitters and various peptide hormones [6,7]. Recent research has shown a role for ascorbate as a cofactor for the dioxygenases involved in gene regulation through modulation of transcription factors and epigenetic marks [4,5]. These latter gene-regulatory functions of ascorbate are responsible for modifying numerous signaling and biosynthetic pathways in the body and thus could play a major role in its observed health and disease modifying effects.

The biological functions of ascorbate depend upon its ability to donate electrons [6]. Loss of one electron generates the ascorbyl radical intermediate, and loss of two electrons generates dehydroascorbate (DHA, which can also be formed via dismutation of the ascorbyl radical) [2]. Detection of DHA in blood samples collected from different patient cohorts has been proposed to be an indication of oxidative stress occurring in these patients [8,9,10,11,12]. However, other investigators have been unable to detect elevated concentrations of DHA in comparable patient cohorts or in smokers, who are known to be under enhanced oxidative stress [13,14,15]. Therefore, there appears to be controversy in the literature. However, ascorbate is extremely sensitive to oxidation and factors such as oxygen, high temperature, UV light, and iron and copper contamination accelerate this process [16]. DHA is also unstable at neutral pH, and is rapidly hydrolysed to 2,3-diketogulonic acid in solution [17]. As such, samples in which ascorbate is to be measured must be very carefully handled and processed to avoid any artefactual oxidation of ascorbate with associated formation of DHA, and/or DHA degradation. The accurate quantification of ascorbate, and by extension DHA, in biological samples is vital to determining its potential health effects.

The current gold standard for the measurement of ascorbate and DHA in biological samples is high performance liquid chromatography (HPLC) with electrochemical detection [18]. Older methods relied upon colourimetric or fluorometric detection of ascorbate or DHA (following complete oxidation of the sample), with variable sensitivities and specificities [18]. An acid or alcohol precipitation step is typically incorporated into sample processing to precipitate protein and stabilize the ascorbate and DHA: meta-phosphoric acid (MPA), trichloroacetic acid (TCA), perchloric acid (PCA) and methanol have all been used. They are often combined with a metal chelator such as ethylenediaminetetraacetic acid (EDTA) or diethylene-triaminepentaacetic acid (DTPA), to help prevent ex vivo ascorbate oxidation [18]. The type of anticoagulant used for blood sample collection may also impact on recovery of ascorbate, with EDTA and heparin tubes providing the most accurate measurement of both ascorbate and DHA [19].

Due to the apparent controversy in the literature surrounding concentrations of DHA in clinical samples, we herein report on the measurement of DHA in a selection of different clinical cohorts (diabetes, pneumonia, cancer, and critically ill) using HPLC with electrochemical detection. We have also determined both the short-term and long-term stability of ascorbate in clinical samples treated under different conditions in order to better elucidate how blood samples should be handled and processed by investigators for accurate ascorbate and DHA assessment.

## 2. Materials and Methods 

### 2.1. In Vitro Stability of Ascorbate and DHA

DHA is a dimer in the crystalline state and can be difficult to fully solubilise. Thus, the soluble DHA concentration was confirmed spectrophotometrically at 265 nm following reduction with 2.5 mmol/L dithiothreitol for 5 min at room temperature (ε = 14500 M^−1^ cm^−1^). The in vitro stability of ascorbate (Sigma-Aldrich, Auckland, New Zealand) and DHA (Sigma-Aldrich, Auckland, New Zealand) in phosphate-buffered saline (PBS) was assessed spectrophotometrically. Fifty micromolar ascorbate or DHA was dissolved in PBS in the presence and absence of DTPA. Samples were removed at indicated time points and the absorbance at 265 nm determined (ε = 14500 M^−1^ cm^−1^). Samples were blanked against buffer containing DTT when required.

### 2.2. Stability of Ascorbate in Blood and Plasma

Blood was obtained from healthy volunteers at the University of Otago, Christchurch. Ethical approval was granted from the Upper South A Regional Ethics Committee (URA/10/03/021) and all donors provided written informed consent. Blood samples were collected into EDTA or lithium heparin anticoagulant tubes as indicated. To prepare plasma, samples were centrifuged at 1000× *g* for 10 min at 4 °C. Plasma was used immediately or alternatively stored at −80 °C until required.

The short-term stability of ascorbate and DHA in whole blood and isolated plasma was determined following collection into sterile EDTA or heparin vacutainer tubes in a sufficient amount for 24 h experiments. Following sterile distribution, tubes of blood or plasma were incubated in the dark, on ice, at 4 °C or at room temperature, and at indicated time points a sample was removed for processing for ascorbate HPLC analysis.

### 2.3. Long-Term Stability of Ascorbate and DHA

The long-term stability of ascorbate and DHA at −80 °C was assessed. Triplicates of standard DHA (~50 µmol/L) were prepared in PBS, aliquoted into cryovials and stored at −80 °C. Similarly triplicates of plasma and PCA-extracts of plasma were aliquoted into cryovials and stored at −80 °C. Triplicates of each sample were removed monthly for one year and ascorbate analysis undertaken. Analysis of the DHA samples required reduction with tris(2-carboxyethyl)phosphine hydrochloride (TCEP) prior to HPLC analysis. 

### 2.4. Detection of DHA in Clinical Samples

Clinical samples which had been collected for vitamin C observational and interventional studies and which had had ascorbate analysis carried out in the presence and absence of the reducing agent TCEP were included in the current analysis. These studies included samples that were processed for ascorbate HPLC analysis prior to storage: prediabetes cohort (*n* = 24, ACTRN12616000858493), colorectal cancer cohort (*n* = 30, ACTRN12615001277538), community acquired pneumonia cohort (*n* = 24) and haematological cancer cohort (*n* = 6, HDEC16STH235), and critically ill surgical cohort (*n* = 17) and septic shock cohort (*n* = 24) [20]; or samples that were stored as frozen plasma: community cohort (*n* = 20) [21], and normal glucose control cohort (*n* = 32), prediabetes cohort (*n* = 24), and type 2 diabetes mellitus cohort (*n* = 30) [22]. All clinical samples were collected into EDTA anticoagulant tubes, processed at 4 °C, and stored at −80 °C prior to HPLC analysis.

### 2.5. Sample Preparation for HPLC Analysis

An aliquot of fresh (or previously frozen) EDTA or heparin plasma was treated with an equal volume of ice-cold 0.54 mol/L HPLC-grade perchloric acid (PCA) solution containing 100 µmol/L of the metal chelator diethylenetriaminepentaacetic acid (DTPA) to precipitate proteins and stabilize the ascorbate [23]. Samples were vortexed, incubated on ice for a few minutes, then centrifuged at 4 °C to remove precipitate. Samples were further diluted with an equal volume of ice cold 77 mmol/L PCA solution containing 100 µmol/L DTPA prior to HPLC analysis. 

TCEP is a strong reducing agent that has recently been shown to reduce DHA to ascorbate at low pH [24,25]. To confirm that TCEP would reduce DHA using our extraction conditions with PCA, concentration and time-dependent experiments were undertaken with standard DHA. We found that TCEP was an effective reductant using the following conditions: 32 mmol/L for 3 h on ice (data not shown). Thus, a 100 µL aliquot of the PCA-supernatant was treated with 10 µL of TCEP (100 mg/mL stock) for 3 h at 4 °C. Samples were further diluted with an equal volume of 77 mmol/L PCA solution containing 100 µmol/L DTPA prior to HPLC analysis. DHA is calculated as the difference between total vitamin C (with TCEP) and ascorbate values (without TCEP).

### 2.6. Ascorbate HPLC Analysis

The ascorbate content of the samples was determined by reverse phase HPLC with electrochemical detection [23]. Samples were separated on a Synergi 4 µ Hydro-RP 80 A column 150 × 4.6 mm (Phenomenex NZ Ltd., Auckland, New Zealand) using an Ultimate 3000 HPLC unit with an Ultimate 3000 ECD-3000RS electrochemical detector and a Model 6011RS coulometric cell. The settings were as follows: +250 mV analytical electrode potential; +300 mV guard cell potential; autosampler chilled to 4 °C and column temperature set to 30 °C. The mobile phase consisted of 80 mmol/L sodium acetate buffer, pH 4.8, containing DTPA (0.54 mmol/L) with the ion-pair reagent n-octylamine (1 µmol/L) added just prior to use. The buffer was delivered isocratically at a flow rate of 1.2 mL/min and 20 µL of sample injected. A standard curve of sodium-l-ascorbate, standardised spectrophotometrically at 245 nm (ε = 9860 M^−1^ cm^−1^), was freshly prepared for each HPLC run in 77 mmol/L HPLC-grade PCA containing DTPA (100 µmol/L). Plasma ascorbate concentration is expressed as µmol/L.

Control experiments were carried out to determine the stability of PCA/DTPA extracts in the HPLC autosampler chilled to 4 °C (*n* = 4–5). Injections were performed once per hour over 24 h. In the absence of the reducing agent TCEP, PCA extracts of both EDTA and heparin plasma were stable for up to 16 h (with a >10% loss observed from this time, data not shown). In comparison, ascorbate was stable for the entire 24 h tested in the presence of TCEP (data not shown). 

### 2.7. Statistical Analyses

Data is represented as the mean ± SD for the clinical samples and mean ± SEM for the in vitro experiments, with the *p* value for statistical significance set at 0.05. Linear regression analysis was used to determine stability of ascorbate and DHA in long-term storage experiments. For short-term stability experiments, statistical difference from time zero was determined by repeated measures one-way analysis of variance with Dunnett’s test for multiple comparison. Statistical analyses were carried out using GraphPad Prism version 7.03 (La Jolla, CA, USA).

## 3. Results

### 3.1. Detection of DHA in Clinical Samples

Analysis of clinical samples in the presence and absence of the reducing agent TCEP allowed us to quantify the amount of DHA present through subtraction of ascorbate (without TCEP) from total vitamin C (with TCEP). Table 1 shows concentrations of DHA in plasma samples from various patient cohorts that were processed and stabilized with PCA and DTPA prior to storage at −80 °C. DHA concentrations were very low to negligible. Only samples that exhibited haemolysis contained appreciable amounts of DHA (Table 1), likely due to ex vivo release of iron from haemoglobin during sample processing (protein precipitation step) and subsequent metal-ion dependent oxidation of ascorbate (see in vitro experiments below).

In contrast, plasma samples that had been stored at −80 °C prior to processing for ascorbate HPLC analysis exhibited ~10-fold higher concentrations of DHA than samples processed and stabilized with PCA and DTPA prior to storage (Table 2). Long-term storage experiments indicated that DHA was relatively stable at −80 °C for up to 1 year, with <8% loss over this time (*p* = 0.097). This indicates that if any ascorbate is oxidized to DHA in plasma during sample processing and/or storage, due to the relative stability of DHA at −80 °C, it should be possible to recover the oxidized ascorbate using TCEP prior to HPLC analysis. As our other long-term storage experiments indicated that ascorbate is stable at −80 °C for at least 1 year, both as plasma (94% remaining at 52 weeks, *p* = 0.562) and PCA/DTPA extracts (101% remaining at 52 weeks, *p* = 0.572), this indicates that the DHA present in the stored plasma samples may have been generated during handling of the plasma samples prior to storage. In support of this, the amount of DHA in the clinical samples did not differ depending on how long the samples had been stored (from 4 months to 2 years, data not shown).

### 3.2. In Vitro Stability of Ascorbate and DHA

**Figure 1:**
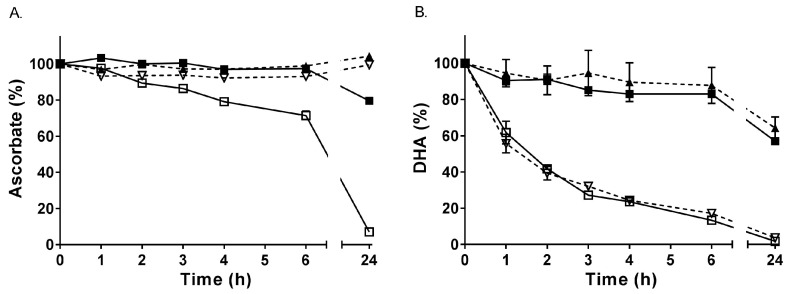
Stability of ascorbate and DHA in PBS. (**A**) Ascorbate (50 µmol/L) was incubated on ice (■,▲) or at room temperature (☐,▽) in the absence (solid line) or presence (dashed line) of the metal chelator DTPA (100 µmol/L). (**B**) DHA (50 µmol/L) was incubated on ice (■,▲) or at room temperature (☐,▽) in the absence (solid line) or presence (dashed line) of DTPA (100 µmol/L). Samples were analysed for ascorbate spectrophotometrically (following reduction of DHA with DTT) at the indicated time points. Data represent the mean ± SEM (*n* = 3). Repeated measure one-way ANOVA with Dunnett’s test for multiple comparison showed no significant loss of ascorbate on ice over 6 h (*p* = 0.697), but a significant decrease at room temperature from 2 h (in the absence of DTPA, *p* = 0.030). A small but significant decrease in DHA on ice was observed from 3 h (*p* = 0.042) and a large decrease in DHA at room temperature was observed from 1 h (*p* = 0.068).

Ascorbate in buffer is stable for at least 6 h if kept on ice (or at 4 °C), with a small decrease in concentration observed at 24 h (Figure 1A). In contrast, ascorbate was much less stable when incubated at room temperature, with a statistically significant decrease from 2 h onwards (*p* < 0.05). The presence of the metal chelator DTPA prevented the loss of ascorbate, indicating that the loss observed at room temperature is due to metal ion-dependent oxidation of ascorbate by contaminating transition metal ions in the buffer. When the same experiment was undertaken with DHA in buffer, the concentration of DHA decreased over time at room temperature, with a half-life of about 1.5 h, but was relatively stable on ice for up to 6 h (Figure 1B). Addition of DTPA to the DHA solution did not inhibit its degradation, which occurs via hydrolysis rather than metal ion-dependent oxidation. As mentioned above, long term storage experiments indicated that DHA was relatively stable at −80 °C for at least 1 year, likely due to attenuation of hydrolysis reactions at this temperature.

### 3.3. Stability of Ascorbate during Sample Collection and Processing

#### 3.3.1. Stability in Whole Blood

**Figure 2:**
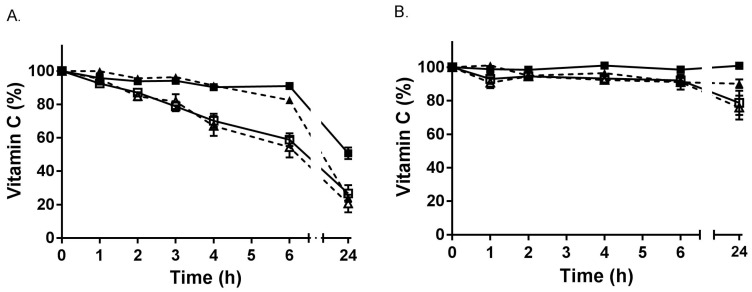
Stability of vitamin C in whole blood. (**A**) EDTA-blood was incubated on ice (■,▲) or at room temperature (☐,△). (**B**) Heparin-blood was incubated on ice (■,▲) or at room temperature (☐,△). Samples without TCEP reduction are shown with dashed lines, and those with TCEP using solid lines. Samples were extracted for ascorbate HPLC analysis at the indicated time points. Data represent the mean ± SEM (*n* = 3). Repeated measure one-way ANOVA with Dunnett’s test for multiple comparison indicated no significant decrease in ascorbate in EDTA-blood on ice over 6 h (*p* = 0.153), but a significant decrease in ascorbate at room temperature from 2 h (*p* = 0.046). There was no significant decrease in ascorbate in heparin-blood on ice for 24 h (*p* = 0.182), or at room temperature over 6 h (*p* = 0.163).

Blood samples drawn into EDTA and heparin tubes were incubated for up to 24 h on ice or at room temperature (Figure 2). Ascorbate was stable for at least 6 h in both EDTA and heparin tubes when kept on ice (or at 4 °C). However, a time-dependent decrease in ascorbate was observed in EDTA blood when incubated at room temperature, with a significant decrease observed from 2 h onwards (*p* < 0.05). This loss could not be recovered via reduction of the samples with TCEP, likely due to the short half-life of DHA at room temperature. 

#### 3.3.2. Stability in Plasma

**Figure 3:**
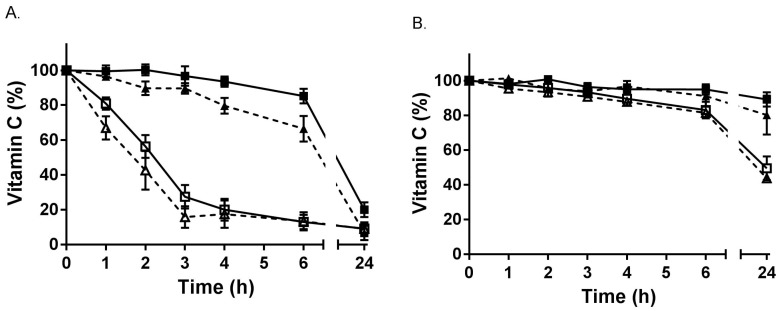
Stability of vitamin C in plasma. (**A**) EDTA-plasma was incubated on ice (■,▲) or at room temperature (☐,△). (**B**) Heparin-plasma was incubated on ice (■,▲) or at room temperature (☐,△). Samples without TCEP are shown with dashed lines, and those with TCEP using solid lines. Samples were extracted for ascorbate HPLC analysis at indicated time points. Data represent the mean ± SEM (*n* = 3–7). Repeated measure one-way ANOVA with Dunnett’s test for multiple comparison showed a significant decrease in ascorbate in EDTA-plasma on ice from 4 h (in the absence of TCEP, *p* = 0.016), and at room temperature from 1 h (*p* = 0.026). There was no significant decrease in ascorbate in heparin-plasma on ice for 24 h (*p* = 0.544), or at room temperature over 6 h (*p* = 0.396).

Plasma isolated from EDTA and heparin blood samples was incubated for up to 24 h on ice or at room temperature (Figure 3). Although ascorbate was relatively stable for the first few hours when EDTA plasma was kept on ice (or at 4 °C), a time-dependent decrease of ascorbate was observed at room temperature, with approximately 50% lost by 2 h. In comparison, ascorbate was more stable in heparin-plasma, with only a small drop in ascorbate observed over time when the sample was kept at room temperature (Figure 3B). Once again, the loss of ascorbate at room temperature could not be recovered following incubation with TCEP.

## 4. Discussion

We have shown in a selection of different clinical cohorts that DHA can be detected in only very low micromolar amounts, if at all. All of the clinical samples were collected into EDTA anticoagulant tubes and were kept on ice/at 4 °C during sample handling and processing, and most were stabilized with PCA/DTPA prior to analysis or storage. The only samples that contained any appreciable amounts of DHA were those exhibiting haemolysis or those which had been stored as plasma prior to processing for ascorbate analysis. Koshiishi et al. [26] have previously shown that oxidation of ascorbate to DHA can occur following acidic deproteination due to catalysis by ferric ion released from haemoglobin and transferrin. When these investigators measured ascorbate directly, without acid deproteination, no DHA was observed, suggesting that DHA detected in acidified plasma samples is due to artefactual ascorbate oxidation [26]. Our in vitro experiments confirm that loss of vitamin C at physiological pH is due to the presence of contaminating metal ions, as indicated by complete inhibition of this loss with the metal chelator DTPA. This indicates that any samples containing haemolysis will require reduction to recover oxidized ascorbate prior to analysis. Although DHA is unstable at room temperature, exhibiting a half-life of ~1.5 h at physiological pH, we have shown that it is stable for several hours at 4 °C, and also exhibits relatively good long-term stability at −80 °C at physiological pH. Others have shown that, like ascorbate, DHA also has good long-term stability in acidified solutions [27,28]. This indicates that if any ascorbate should become oxidized to DHA during sample handling and processing, due to the relative stability of DHA at −80 °C, it should be possible to recover the oxidized ascorbate using an appropriate reducing agent. 

Our short-term stability experiments in whole blood and plasma samples indicated that although ascorbate was stable for at least several hours at 4 °C, there was clear loss of ascorbate in EDTA whole blood if left at room temperature for 2 h and rapid loss, even within 1 h, in EDTA plasma. EDTA is routinely used as an anticoagulant for clinical samples as it chelates calcium ions which are required for clotting enzymes [29]. EDTA also chelates transition metal ions, such as iron and copper, which can cause oxidation of ascorbate. Therefore, EDTA is often added as a chelating agent to samples requiring measurement of ascorbate. However, EDTA chelates of iron and copper are not redox inactive, in other words, they are still capable of oxidizing ascorbate [30,31]. Although, addition of EDTA can help stabilize ascorbate in acidic solutions at 4 °C [18,32], it is clearly unable to stabilize ascorbate at physiological pH, particularly at room temperature [33]. Therefore, if ascorbate is to be measured in EDTA blood samples, it is essential that these are kept cold at all times during handling, processing and analysis of the samples.

A number of studies have previously emphasised the importance of the collection and processing method for maintaining ascorbate stability in blood samples, with temperature and pH being the most important factors, particularly with respect to the anticoagulant used and perhaps also the acid precipitant, although the latter is less clear [33,34,35,36,37,38,39,40]. Our review of the literature of DHA concentrations reported in clinical cohorts (summarized in Table 3) has also highlighted that high concentrations of DHA are observed in all studies (except one [13]) that have used colourimetric or flourometric methods for detecting DHA [8,9,10,11,12]. In comparison, DHA levels in clinical cohorts are much lower when HPLC with electrochemical detection is used [14,15], regardless of the anticoagulant [19,41] or the precipitating agent used [14,26]. This is likely due to the lack of specificity of the colourimetric/fluorometric methods, which are prone to interference by numerous different compounds in the blood, including transition metal ions [18]. It is noteworthy that depleted vitamin C concentrations, which are typically observed during severe infection, are associated with enhanced haemolysis [42], thus potentially contributing interfering metal ions to the colourimetric/fluorometric assays. Overall, this suggests that DHA may not be present in circulation and detection is due to assay conditions resulting in ex vivo ascorbate oxidation [41]. Furthermore, since DHA is rapidly taken up by cells via their glucose transporters (GLUTs), followed by intracellular reduction to ascorbate via enzymatic and non-enzymatic means [43,44], it would be very surprising indeed to detect appreciable amounts of DHA in the circulation.

Despite the plethora of data on vitamin C stability under various assay conditions, clinical studies have been published utilizing less than ideal handling and processing conditions (e.g., EDTA blood stored at room temperature for up to 5 h followed by colourimetric detection), which results in low concentrations of ascorbate detected (i.e., 32 µmol/L) and a high percentage of deficiency (6% with plasma concentrations <11 µmol/L) [45]. This is in comparison to other age-matched studies where EDTA blood was handled on ice or at 4 °C and analyses were carried out by HPLC with electrochemical detection [21,46]. Similarly, the low serum vitamin C concentrations reported in a cohort of young Canadians (i.e., 24–30 µmol/L with 14% deficiency), despite saturating intakes (i.e., 228–248 mg/d) [47], has been questioned by Hoffer, due to likely issues with sample handling, processing and storage [48]. Thus, due to the numerous different factors that can affect ascorbate stability in clinical samples during handling, processing, storage and analysis, it is vital to assess ascorbate stability under the specific conditions being used in each study.

## 5. Conclusions

Our study and accompanying literature review have highlighted a number of important points with respect to accurate analysis of ascorbate and DHA in clinical study samples:

- Plasma stored at −80 °C prior to processing for ascorbate analysis contains variable amounts of DHA, requiring treatment with a reducing agent prior to ascorbate analysis (DHA is relatively stable for at least a year at −80 °C at physiological pH).
- Haemolysis facilitates oxidation of ascorbate, likely due to the release of catalytic iron from haemoglobin following acid precipitation, requiring reduction of samples prior to ascorbate analysis (DHA is stable for at least five years at −80 °C under acidic conditions).
- EDTA anticoagulant samples need to be kept cold at all times during handling, processing and analysis as EDTA-chelated iron is redox active at physiological pH and can facilitate ascorbate oxidation via redox cycling.
- Colourimetric/fluorometric ascorbate assays appear to generate high concentrations of DHA via artefactual ex vivo oxidation of ascorbate, regardless of the anticoagulant or deproteinization method used. In comparison, analysis using HPLC with electrochemical detection does not detect appreciable DHA concentrations in clinical samples.

## Figures and Tables

**Table 1:** Dehydroascorbic acid (DHA) concentrations in clinical samples.

Cohort	Description (n)	DHA (µmol/L) ^1^
Community	Prediabetes (22)	2.8 ± 4.2
Infection	Pneumonia (20)	1.2 ± 1.2
Cancer	Colorectal (27)	2.5 ± 3.3
	Haematological (6)	1.0 ± 0.4
Critically ill	Septic shock (24)	0.1 ± 2.2
	Surgical (15)	0.0 ± 1.5
Excluded ^2^	Heamolysed (9)	17 ± 11

^1^ Values represent mean ± SD. ^2^ Haemolysed samples were excluded and presented separately: 2 from prediabetes, 2 from pneumonia, 3 from colorectal cancer, and 2 from surgical cohorts.

**Table 2:** DHA concentrations in stored plasma samples ^1^.

Cohort (n)	DHA (µmol/L)
Community (20)	24 ± 7
Normal glucose control (32)	10 ± 4
Prediabetes (24)	9 ± 5
Type 2 diabetes mellitus (30)	8 ± 6

^1^ Values represent mean ± SD.

**Table 3:** DHA concentrations in published clinical studies.

Cohort (n)	DHA (µmol/L) ^1^	Anticoagulant, Deproteinization, and Detection	Ref.
Controls (28) Pneumonia–died (7) Pneumonia–survived (15) Convalescent (13)	3 ± 1 39 ± 2 23 ± 1 9 ± 1	Oxalate SSA DCPIP	[8]
Controls (10) Viral hepatitis (26) Liver carcinoma (11)	5 ± 2 29 ± 8 32 ± 5	Heparin TCA DNPH	[9]
Controls (20) Rheumatoid arthritis (13)	12 ± 4 22 ± 9	Serum MPA/EDTA PDA/HPLC	[10]
Controls (37) Diabetic–male (25) Diabetic–female (12)	0 12 ± 2 12 ± 2	Heparin MPA DNPH	[11]
Controls (20) Diabetic (27)	2.0 ± 1.5 3.3 ± 3.0	EDTA MPA/oxalate DNPH	[13]
Controls (34) Critically ill (62) Diabetic (24) Gastritis (21)	2.3 (−2.9–5.8) 1.4 (−0.8–2.9) 2.8 (0.0–6.3) 2.3 (0.6–2.9)	Heparin MPA HPLC	[14]
Controls (124) Smokers (82)	0.1 ± 2.4 0.8 ± 2.3	MPA HPLC-ECD	[15]

^1^ Values represent mean ± SD or mean (and range). Abbreviations: SSA, sulfosalicylic acid; TCA, trichloroacetic acid; MPA, metaphosphoric acid; EDTA, ethylenediaminetetraacetic acid; DCPIP, 2,6-dichlorophenol indophenol; DNPH, 2,4-dinitrophenylhydrazine; PDA, 1,2-phenylenediamine; ECD, electrochemical detection.
